# Presence of *Burkholderia pseudomallei* in Soil, Nigeria, 2019

**DOI:** 10.3201/eid2905.221138

**Published:** 2023-05

**Authors:** Jelmer Savelkoel, Rita O. Oladele, Chiedozie K. Ojide, Rebecca F. Peters, Daan W. Notermans, Justina O. Makinwa, Maaike C. de Vries, Marion A.E. Sunter, Sébastien Matamoros, Nasiru Abdullahi, Uche S. Unigwe, Alani S. Akanmu, W. Joost Wiersinga, Emma Birnie

**Affiliations:** Amsterdam UMC location University of Amsterdam, Amsterdam, the Netherlands (J. Savelkoel, D.W. Notermans, S. Matamoros, W.J. Wiersinga, E. Birnie);; Lagos University Teaching Hospital, Lagos, Nigeria (R.O. Oladele, R.F. Peters, J.O. Makinwa, A.S. Akanmu);; College of Medicine University of Lagos, Lagos, Nigeria (R.O. Oladele, A.S. Akanmu);; Ebonyi State University, Abakaliki, Nigeria (C.K. Ojide);; Alex Ekwueme Federal University Teaching Hospital, Abakaliki, Nigeria (C.K. Ojide, U.S. Unigwe);; National Institute for Public Health and the Environment (RIVM), Bilthoven, the Netherlands (D.W. Notermans, M.C. de Vries, M.A.E. Sunter);; Federal Medical Centre, Abuja, Nigeria (N. Abdullahi);; University of Nigeria Teaching Hospital, Enugu, Nigeria (U.S. Unigwe)

**Keywords:** Burkholderia pseudomallei, Burkholderia thailandensis, melioidosis, Nigeria, environmental study, bacteria

## Abstract

Melioidosis, caused by the soil-dwelling bacterium *Burkholderia pseudomallei*, is predicted to be endemic in Nigeria but is only occasionally reported. This report documents the systematic identification of the presence of *B. pseudomallei* and *B. thailandensis* in the soil across multiple states in Nigeria.

The gram-negative, soil-dwelling bacterium *Burkholderia pseudomallei* is the causative agent of melioidosis, which is an important cause of lethal community-acquired sepsis throughout the tropics (*1*). Melioidosis is predicted to be endemic in Nigeria, a country with the highest estimated annual incidence, mortality, and disease burden in Africa, partly explained by its suitable environment and large population (*2*–*4*). Clinical evidence of melioidosis in Nigeria is scarce and based only on traveler-associated cases in the United Kingdom and reports from Nigeria presuming the presence of *B. pseudomallei* (*4*–*7*). This study was a collaborative effort prompted by the African Melioidosis Workshop in Lagos, Nigeria (*4*); our goal was to determine the environmental presence of *B. pseudomallei* in Nigeria. Ethics approval was obtained from the National Health Research Ethics Committee of Nigeria (approval no. NHREC/01/01/2007-26/03/2019).

We performed an environmental soil sampling study based on consensus guidelines for the identification of *B. pseudomallei* (*8*). We consulted local residents and maps to select sites associated with the occurrence of *B. pseudomallei*, as we have done previously (*9*). Using a fixed interval grid and samples taken 5 meters apart, we collected 100 soil samples per site across 8 sites in Nigeria during the rainy season in April–May 2019 (Table; Appendix). We collected a total of 800 samples in the northwestern state Kebbi, southwestern state Ogun, and southeastern states Ebonyi and Enugu. We collected soil at a depth of 65 cm and processed 10 g of soil within 7 days to enable selective enriched culture (*8*,*10*). We screened isolates by using colony morphology and, if results were suspect, used matrix-assisted laser desorption/ionization-time of flight mass spectrometry (MALDI Biotyper Compass v4.1 and Compass Library v10; Bruker Daltonics, https://www.bruker.com). We subjected all presumptive *B. pseudomallei* isolates to real-time multiplex PCR and performed whole-genome sequencing on 9 *B. pseudomallei* isolates and 3 *B. thailandensis* isolates by using the NextSeq 500/550 platform (Illumina, https://www.illumina.com) (Appendix). We then included the same *B. pseudomallei* isolates in our phylogenetic comparison and used them for antimicrobial susceptibility testing (Appendix). Sequences for the samples in this study are available on the European Nucleotide Archive database (project number PRJEB54705, sample accession nos. ERS12451640–51; https://www.ebi.ac.uk/ena/browser/home).

**Table:** Site characteristics and distribution of *Burkholderia pseudomallei* at 8 sampling sites in Nigeria, 2019

Site	Location	State	Place	Site characteristics	Sample holes positive for *B. pseudomallei*
A	Southwestern	Ogun	Lufoko	Rice field, dry	0
B	Southwestern	Ogun	Ige	Rice field, dry	0
C	Northwestern	Kebbi	Birnin Kebbi*	Rice field, moist	4
D	Northwestern	Kebbi	Birnin Kebbi*	Rice field, moist	1
E	Southwestern	Ogun	Sunmoge	Cattle, grassland next to river, moist	0
F	Southeastern	Ebonyi	Abakaliki	Rice field and cassava crops, moist	38
G	Southeastern	Ebonyi	Abakaliki	Rice swamp, wet	1
H	Southeastern	Enugu	Nenwe	Rice field, moist	14

*The sampling sites in Birnin Kebbi were located 3 km apart from each other. An overview of the geographic distribution of sampling sites for *Burkholderia pseudomallei* can be found in the Appendix.

By using the methods described, we isolated *B. pseudomallei* from 58 (7.3%) of 800 samples in 5 (62.5%) of the 8 sampling sites (Table; Appendix). We observed the highest positivity in the southeastern states, with rates as high as 38% in Ebonyi and 14% in Enugu. We also isolated the nonpathogenic *B. thailandensis* from 193 (24.1%) of 800 samples in 4 (50%) of the 8 sampling sites. Antimicrobial susceptibility of the *B. pseudomallei* isolates displayed overall sensitivity against antibiotic agents commonly used for the treatment of melioidosis, such as ceftazidime, meropenem, and trimethoprim/sulfamethoxazole (Appendix). 

We conducted phylogenetic analysis of our 9 sequenced *B. pseudomallei* isolates and 13 additional genomes originating from Africa, all retrieved from the European Nucleotide Archive database. The phylogenetic tree revealed a cluster of predominantly continental African origin that included all of the soil isolates from Nigeria and a cluster of strains derived mainly from the Indian Ocean region (Figure). Our *B. pseudomallei* isolates did not closely match the previously sequenced traveler-associated strain from Nigeria (ERR298772) (*7*): the genome differed by 8,370 to 9,431 core single-nucleotide polymorphisms. We speculated that the higher positivity in the southeastern states reflects the relatively high annual precipitation in southeastern Nigeria as compared with sampling sites in the northwestern and southwestern states (Appendix).

**Figure:**
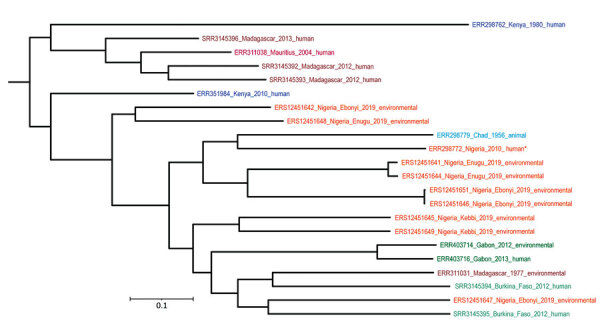
Phylogenetic tree of *Burkholderia pseudomallei* genomes from Nigeria (orange text) and additional genomes originating from Africa, all retrieved from the European Nucleotide Archive database. Tree generated by FastTree (http://www.microbesonline.org/fasttree) based on core single-nucleotide polymorphisms distance and visualized with iTOL (https://itol.embl.de). Colors indicate countries of origin. Asterisk indicates a previously sequenced, traveler-associated strain. Scale bar indicates number of nucleotide substitutions per site.

Adopting a culture-based approach, combined with matrix-assisted laser desorption/ionization-time of flight mass spectrometry, real-time PCR, and whole genome sequencing allowed us to identify the environmental presence of *B. pseudomallei*. Limitations of our study include possible sampling errors and false-negative samples because we relied on a culture-based approach instead of using an additional quantitative PCR on soil samples (*9*). Moreover, we did not collect soil samples in multiple seasons to investigate a seasonal pattern, nor did we collect water or air samples.

In conclusion, we documented the systematic confirmation of the environmental presence of *B. pseudomallei* and *B. thailandensis* across multiple states in Nigeria. We identified the highest *B. pseudomallei* positivity rates in the southeastern states Ebonyi and Enugu. Phylogenetic analysis clustered our *B. pseudomallei* isolates with previous genomes that originated mostly from continental Africa. Our results highlight the probability of unrecognized melioidosis in Nigeria and warrant the attention of health workers and public health officials. Improving capacity and increasing awareness, together with environmental, serologic, and disease surveillance, is needed to increase our understanding of the melioidosis burden within Nigeria.

Appendix Additional information for presence of *Burkholderia pseudomallei* in soil, Nigeria, 2019.
