# Supplementary material for: Presence of Burkholderia pseudomallei in Soil, Nigeria, 2019
Source: Emerg Infect Dis. 2023 May;29(5):1073–5. doi: 10.3201/eid2905.221138 (PMC10124662; doi:10.3201/eid2905.221138)
Supplement: Appendix — Additional information for presence of Burkholderia pseudomallei in soil, Nigeria, 2019. [file 22-1138-Techapp-s1.pdf]

Article DOI: http://doi.org/10.3201/eid2905.221138

EID cannot ensure accessibility for supplementary materials supplied by authors. Readers who have difficulty accessing supplementary content should contact the authors for assistance.

Presence of *Burkholderia pseudomallei* in Soil, Nigeria, 2019

Appendix

Climatological Data

Appendix Table 1. Annual mean temperature and precipitation of sampled states in Nigeria (1991–2020)

Location	State	Annual mean temperature (°C)	Annual precipitation (mm)
Northwestern	Kebbi	28.71	849.46
Southwestern	Ogun	27.55	1367.16
Southeastern	Enugu	26.85	1822.48
Southeastern	Ebonyi	26.88	1966.70

Annual mean temperature and precipitation data of Nigeria (1991–2020) is derived from the Climate Change Knowledge Portal of the World Bank Group (*1*).

Polymerase Chain Reaction

We performed a TaqMan real-time multiplex polymerase chain reaction (RT-PCR) assay on all presumptive *B. pseudomallei* isolates based on colony morphology and matrix assisted laser desorption/ionization-time of flight mass spectrometry (Bruker Daltonics). We optimized a previously described RT-PCR assay with several modifications as discussed hereafter (*2*). The gene targets were as follows: *Orf11* for *B. pseudomallei*, a hypothetical 16.5 kDa protein for *B. mallei*, and *fliC* for *B. pseudomallei*, *B. mallei*, and *B. thailandensis*. The species-specific primers were modified to optimize the reaction (**Appendix Table 2**). The reaction volume of 20 µL consisted of 10 µL SensiFast master mix (Bioline), 5 µL primers-probes mix, and 5 µL of bacterial DNA. We extracted DNA of five colonies in 100 µL AE-buffer (Qiagen) at 100°C for 30 minutes. Bacterial lysates of several colonies were diluted a 100-fold with TE-buffer before being added to the final reaction volume. The primers-probes mix was prepared in TE-buffer to a final concentration of 2 pmol/µl for primers and 0.4 pmol/µl for probes. We included a positive control that consisted of a mix of *B. pseudomallei* and *B. mallei*, a no template control, and an

internal control using the nucleotide sequence of the seal herpesvirus type 1 (PhHV) to check for inhibition of the reaction (*3*). The PCR reaction was run on a LightCycler 480 II (Roche) with a denaturation cycle of 5 minutes at 95°C followed by 45 amplification cycles of 10 seconds at 95°C and 30 seconds at 60°C. An isolate was considered positive when the exponential phase was reached.

Appendix Table 2. Overview of primers and probes of the multiplex RT-PCR for *Burkholderia* species

Target species	Target gene	Nucleotide sequence	Fluorescent label
B. pseudomallei	*Orf11* forward	5′- ACA AGT GGC CCT ATG GAT TG −3′	
	Orf11 reverse	5′- TCG GTT TCG AAT AAC GGG TA −3′	
	Orf11 probe	5′- ACG ATC TCC GAG AAC GCA CTG AAC A −3′	FAM-BHQ1
B. mallei	16.5 kDa forward	5′- CGA GCT CAG CAA CCT CGT TA −3′	
	16.5 kDa reverse	5′- CGC GGT CTA CCT TGC ATA TT −3′	
	16.5 kDa probe	5′- CAG TAT CCA GGT TTC ACC GCG CTC GAC −3′	Texas Red-BHQ1
B. pseudomallei, *B. mallei*, and *B. thailandensis*	*fliC* forward	5′- GTC AAC AAI CTG CAG GCA AC −3′	
	fliC reverse	5′- CGG TTT CCT GAG IAA AGT CC −3′	
	fliC probe	5′- GGC TCG AAC AAC CTC GCG CAR G −3′	ATTO532-BHQ1
PhHV	PhHV forward	5′- GGG CGA ATC ACA GAT TGA ATC −3′	
	PhHV reverse	5′- GCG GTT CCA AAC GTA CCA A −3′	
	PhHV probe	5′- TTT TTA TGT GTC CGC CAC CAT CTG GAT C −3′	Cy5-BHQ2

Whole-Genome Sequencing

We performed whole-genome sequencing (WGS) on nine *B. pseudomallei* and three *B. thailandensis* isolates using the following methods. We extracted DNA of a loopful of bacteria in 100 µL AE-buffer at 100°C for 30 minutes. Sequences were obtained using the NextSeq 500/550 platform (Illumina). After demultiplexing, low-quality reads were discarded and adaptor sequences were trimmed using Trimmomatic v0.39 (*4*). High quality reads were used for de novo assembly using SKESA v2.4.0 (*5*). Contigs smaller than 500 bp were discarded for the following analyses. Quality of the assemblies was assessed using Quast v5.0.2 (*6*). The sequencing depth was determined by mapping the raw sequencing reads of each isolate to their respective final assembly contigs using minimap2 v2.17 (*7*), then calculating the genome coverage using SAMtools v1.14 and BEDtools v2.30.0 (*8*,*9*). SNP analysis was performed using kSNP3 v3.1.2 and visualized using FastTree v2.0 and iTOL v6 (https://itol.embl.de/) (*10*–*12*). Species identification was confirmed based on WGS data using the software KmerFinder v3.0.2 of the Center for Genomic Epidemiology (https://cge.food.dtu.dk/services/KmerFinder/) (*13*–*15*).

Based on WGS analysis, we phylogenetically characterized nine *B. pseudomallei* isolates. Additional *B. pseudomallei* genomes originating from Africa with known countries were identified via the literature and downloaded from the European Nucleotide Archive database (https://www.ebi.ac.uk/ena/browser/home) (*16*,*17*). We excluded the *B. thailandensis* isolates from further phylogenetic comparisons.

Antimicrobial Susceptibility Testing

We performed antimicrobial susceptibility testing on the nine sequenced *B. pseudomallei* isolates following the guidelines for *B. pseudomallei* of the European Committee on Antimicrobial Susceptibility Testing (EUCAST) (*18*–*20*). We used the following antibiotic disks (Becton Dickinson) with disk content between parenthesis: amoxicillin-clavulanic acid (20–10 µg), ceftazidime (10 µg), imipenem (10 µg), meropenem (10 µg), tetracycline (30 µg), chloramphenicol (30 µg), trimethoprim-sulfamethoxazole (1.25–23.75 µg), and gentamicin (10 µg). Of note: gentamicin is not included in the recommendations of EUCAST but was included as an additional control measure as we used Ashdown's selective agar for bacterial isolation. Up to four disks were placed per Mueller-Hinton (MHE) agar plate (bioMérieux). Isolates with unexpected antibiotic resistance were subjected to gradient strip testing (Liofilchem) to establish the minimal inhibitory concentration (MIC) following the previously described methods. Quality control was included as per EUCAST's instructions.

Antimicrobial susceptibility of sequenced *B. pseudomallei* isolates displayed overall sensitivity against antibiotic agents commonly used for treatment of melioidosis, such as ceftazidime, meropenem, and trimethoprim-sulfamethoxazole (**Appendix Table 3**). However, using the disk diffusion method unexpected antimicrobial resistance was observed against meropenem in one *B. pseudomallei* isolate. MIC testing using gradient strips did not confirm meropenem resistance as an MIC of 2 mg/L was observed (breakpoint resistance: >2 mg/L).

Appendix Table 3. Antimicrobial susceptibility of *Burkholderia pseudomallei* isolates following EUCAST's disk diffusion methods

Susceptibility testing				EUCAST breakpoints	
**Antibiotic agent**	**Disk content (µg)**	**Sensitive isolates**	**Zone diameter range (mm)**	**S ≥(mm)**	**R <(mm)**
Amoxicillin-clavulanic acid	20–10	9/9	25–30	50	22
Ceftazidime	10	9/9	22–28	50	18
Imipenem	10	9/9	31–37	29	29
Meropenem*	10	8/9*	22–31	24	24
Tetracycline*	30	9/9	26–34	23	23
Chloramphenicol	30	9/9	26–30	50	22
Trimethoprim-sulfamethoxazole	1.25–23.75	9/9	30–42	50	17
Gentamicin*	10	0/9	0–0	NA	NA

*Meropenem resistance was not confirmed using a minimal inhibitory concentration test. Tetracycline is used to screen for doxycycline susceptibility. Gentamicin is not included in EUCAST's breakpoints for *B. pseudomallei*.
Abbreviations: S = sensitive, R = resistant, NA = not applicable.

Multi Locus Sequence Typing

Multi locus sequence typing (MLST) was performed on the nine sequenced *B. pseudomallei* isolates using the software MLST v2.0.9 of the Center for Genomic Epidemiology (https://cge.food.dtu.dk/services/MLST/) (**Appendix Table 4**) (*21*). Raw sequencing reads were used as data input and the minimum depth for an allele was set at five times. Information on loci and gene function used in the *B. pseudomallei* MLST scheme can be found elsewhere (*22*). Next, the *B. pseudomallei* PubMLST curator and database were consulted to assign sequence types (STs) and to resolve any queries regarding imperfect matches or novel alleles (https://pubmlst.org/organisms/burkholderia-pseudomallei) (*23*).

Appendix Table 4. Multi locus sequence typing of *Burkholderia pseudomallei* isolates: sequence types and allelic profiles

			Allelic profile						
**Sample ID**	**ENA accession number**	**ST**	**ace**	**gltB**	**gmhD**	**lepA**	**lipA**	**narK**	**ndh**
BpsC1	ERS12451645	930	1	1	3	2	5	1	1
BpsC2*	ERS12451649	12*	1	1	13	1	5	1*	1
BpsF1	ERS12451642	1720	1	1	3	2	5	2	1
BpsF2	ERS12451647	12	1	1	13	1	5	1	1
BpsF3	ERS12451651	2023	1	1	19	4	1	2	3
BpsG1	ERS12451646	2023	1	1	19	4	1	2	3
BpsH1	ERS12451644	2024	1	1	3	1	5	152	1
BpsH2*	ERS12451648	Unknown*	1	12	3	1	1	1*	1
BpsH3	ERS12451641	2026	1	1	10	1	5	149	1

*Imperfect narK hit so ST cannot be trusted. Allelic profiles of isolates with an imperfect hit were not uploaded to the PubMLST database.
Abbreviations: ENA = European Nucleotide Archive, ST = sequence type.

References

1. World Bank Group. Climate Change Knowledge Portal [cited 2022 7 June]. https://climateknowledgeportal.worldbank.org/country/nigeria/climate-data-historical.

2. Lowe CW, Satterfield BA, Nelson DB, Thiriot JD, Heder MJ, March JK, et al. A quadruplex real-time PCR assay for the rapid detection and differentiation of the most relevant members of the B. pseudomallei complex: B. mallei, B. pseudomallei, and B. thailandensis. PLoS One. 2016;11:e0164006. PubMed https://doi.org/10.1371/journal.pone.0164006

3. Niesters HG. Quantitation of viral load using real-time amplification techniques. Methods. 2001;25:419–29. PubMed https://doi.org/10.1006/meth.2001.1264

4. Bolger AM, Lohse M, Usadel B. Trimmomatic: a flexible trimmer for Illumina sequence data. Bioinformatics. 2014;30:2114–20. PubMed https://doi.org/10.1093/bioinformatics/btu170

5. Souvorov A, Agarwala R, Lipman DJ. SKESA: strategic k-mer extension for scrupulous assemblies. Genome Biol. 2018;19:153. PubMed https://doi.org/10.1186/s13059-018-1540-z

6. Gurevich A, Saveliev V, Vyahhi N, Tesler G. QUAST: quality assessment tool for genome assemblies. Bioinformatics. 2013;29:1072–5. PubMed https://doi.org/10.1093/bioinformatics/btt086

7. Li H. Minimap2: pairwise alignment for nucleotide sequences. Bioinformatics. 2018;34:3094–100. PubMed https://doi.org/10.1093/bioinformatics/bty191

8. Li H, Handsaker B, Wysoker A, Fennell T, Ruan J, Homer N, et al.; 1000 Genome Project Data Processing Subgroup. The Sequence Alignment/Map format and SAMtools. Bioinformatics. 2009;25:2078–9. PubMed https://doi.org/10.1093/bioinformatics/btp352

9. Quinlan AR, Hall IM. BEDTools: a flexible suite of utilities for comparing genomic features. Bioinformatics. 2010;26:841–2. PubMed https://doi.org/10.1093/bioinformatics/btq033

10. Gardner SN, Slezak T, Hall BG. kSNP3.0: SNP detection and phylogenetic analysis of genomes without genome alignment or reference genome. Bioinformatics. 2015;31:2877–8. PubMed https://doi.org/10.1093/bioinformatics/btv271

11. Price MN, Dehal PS, Arkin AP. FastTree 2—approximately maximum-likelihood trees for large alignments. PLoS One. 2010;5:e9490. PubMed https://doi.org/10.1371/journal.pone.0009490

12. Letunic I, Bork P. Interactive Tree Of Life (iTOL) v5: an online tool for phylogenetic tree display and annotation. Nucleic Acids Res. 2021;49(W1):W293–6. PubMed https://doi.org/10.1093/nar/gkab301

13. Clausen PTLC, Aarestrup FM, Lund O. Rapid and precise alignment of raw reads against redundant databases with KMA. BMC Bioinformatics. 2018;19:307. PubMed https://doi.org/10.1186/s12859-018-2336-6

14. Hasman H, Saputra D, Sicheritz-Ponten T, Lund O, Svendsen CA, Frimodt-Møller N, et al. Rapid whole-genome sequencing for detection and characterization of microorganisms directly from clinical samples. J Clin Microbiol. 2014;52:139–46. PubMed https://doi.org/10.1128/JCM.02452-13

15. Larsen MV, Cosentino S, Lukjancenko O, Saputra D, Rasmussen S, Hasman H, et al. Benchmarking of methods for genomic taxonomy. J Clin Microbiol. 2014;52:1529–39. PubMed https://doi.org/10.1128/JCM.02981-13

16. Chewapreecha C, Holden MT, Vehkala M, Välimäki N, Yang Z, Harris SR, et al. Global and regional dissemination and evolution of Burkholderia pseudomallei. Nat Microbiol. 2017;2:16263. PubMed https://doi.org/10.1038/nmicrobiol.2016.263

17. Stone NE, Hall CM, Browne AS, Sahl JW, Hutton SM, Santana-Propper E, et al. Burkholderia pseudomallei in Soil, US Virgin Islands, 2019. Emerg Infect Dis. 2020;26:2773–5. PubMed https://doi.org/10.3201/eid2611.191577

18. The European Committee on Antimicrobial Susceptibility Testing. Breakpoint tables for interpretation of MICs and zone diameters. Version 12.0, 2022. http://www.eucast.org.

19. The European Committee on Antimicrobial Susceptibility Testing. Routine and extended internal quality control for MIC determination and disk diffusion as recommended by EUCAST. Version 12.0, 2022. http://www.eucast.org.

20. Dance DAB, Wuthiekanun V, Baird RW, Norton R, Limmathurotsakul D, Currie BJ. Interpreting Burkholderia pseudomallei disc diffusion susceptibility test results by the EUCAST method. Clin Microbiol Infect. 2021;27:827–9. PubMed https://doi.org/10.1016/j.cmi.2021.02.017

21. Larsen MV, Cosentino S, Rasmussen S, Friis C, Hasman H, Marvig RL, et al. Multilocus sequence typing of total-genome-sequenced bacteria. J Clin Microbiol. 2012;50:1355–61. PubMed https://doi.org/10.1128/JCM.06094-11

22. Godoy D, Randle G, Simpson AJ, Aanensen DM, Pitt TL, Kinoshita R, et al. Multilocus sequence typing and evolutionary relationships among the causative agents of melioidosis and glanders, Burkholderia pseudomallei and Burkholderia mallei. J Clin Microbiol. 2003;41:2068–79. PubMed https://doi.org/10.1128/JCM.41.5.2068-2079.2003

23. Jolley KA, Bray JE, Maiden MCJ. Open-access bacterial population genomics: BIGSdb software, the PubMLST.org website and their applications. Wellcome Open Res. 2018;3:124. PubMed https://doi.org/10.12688/wellcomeopenres.14826.1

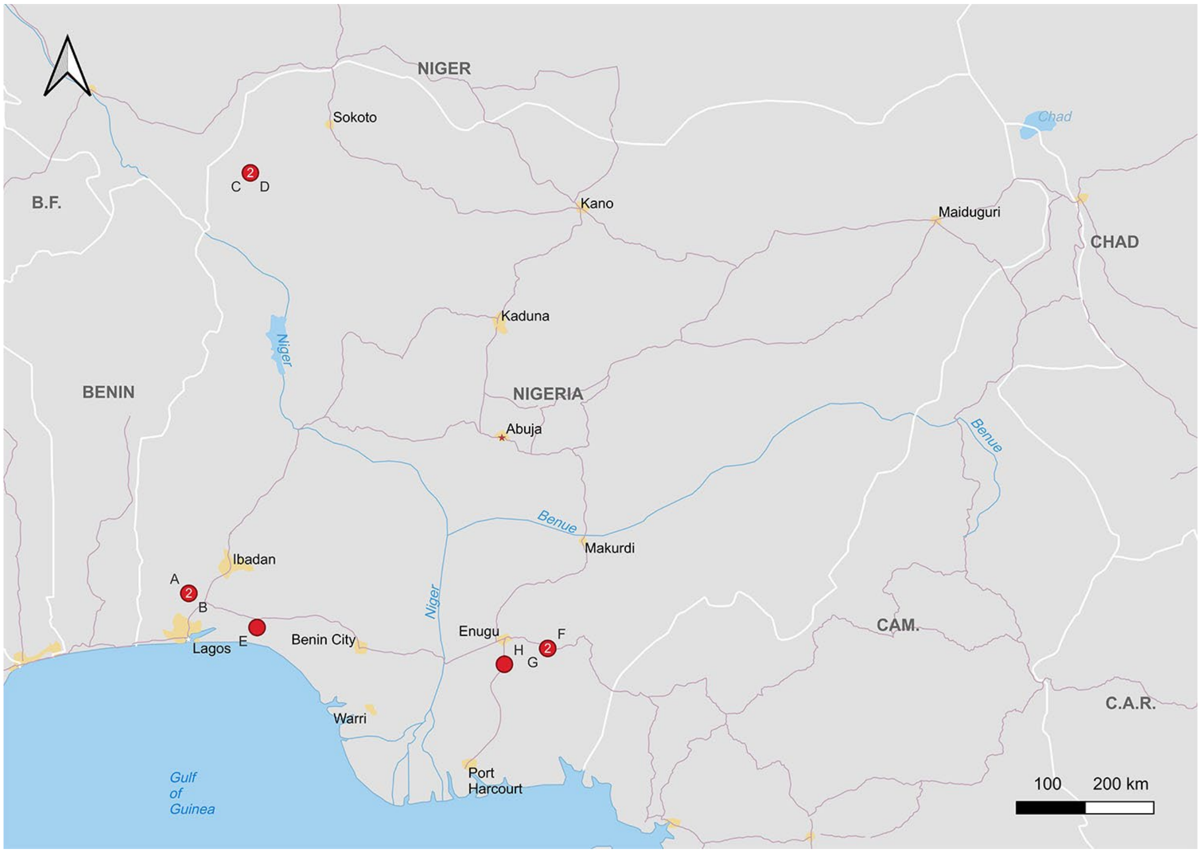

Appendix Figure. Geographic distribution of sampling sites for *Burkholderia pseudomallei* in Nigeria, 2019. Numbers indicate multiple sampling sites. Made with QGIS using Natural Earth data.
